# Ketamine Within Clinically Effective Range Inhibits Glutamate Transmission From Astrocytes to Neurons and Disrupts Synchronization of Astrocytic SICs

**DOI:** 10.3389/fncel.2019.00240

**Published:** 2019-06-11

**Authors:** Yu Zhang, Sisi Wu, Liwei Xie, Shouyang Yu, Lin Zhang, Chengxi Liu, Wenjing Zhou, Tian Yu

**Affiliations:** ^1^Department of Anesthesiology, Affiliated Hospital of Zunyi Medical University, Guizhou, China; ^2^The Key Laboratory of Anesthesia and Organ Protection, Zunyi Medical University, Guizhou, China; ^3^The Key Laboratory of Brain Science, Zunyi Medical University, Guizhou, China

**Keywords:** ketamine, slow inward current, astrocyte, GluN2B receptor, neuromodulation

## Abstract

**Background:**

Astrocytes are now considered as crucial modulators of neuronal synaptic transmission. General anesthetics have been found to inhibit astrocytic activities, but it is not clear whether general anesthetics within the clinical concentration range affects the astrocyte-mediated synaptic regulation.

**Methods:**

The effects of propofol, dexmedetomidine, and ketamine within clinically effective ranges on the slow inward currents (SICs) were tested by using the whole-cell recording in acute prefrontal cortex (PFC) slice preparations of rats. Astrocytes culture and HPLC were used to measure the effects of different anesthetics on the glutamate release of astrocytes.

**Results:**

Propofol and dexmedetomidine showed no significant effect on the amplitude or frequency of SICs. Ketamine was found to inhibit the frequency of SICs in a concentration-dependent manner. The SICs synchronization rate of paired neurons was inhibited by 30 μM ketamine (from 42.5 ± 1.4% to 9.6 ± 0.8%) and was abolished by 300 μM ketamine. The astrocytic glutamate release induced by DHPG, an agonist of astrocytic type I metabotropic glutamate receptors, was not affected by ketamine, and ifenprodil, a selective antagonist of GluN1/GluN2B receptor, blocked all SICs and enhanced the inhibitory effect of 30 μM ketamine on the frequency of SICs. Ketamine at low concentration (3 μM) could inhibit the frequency of SICs, not the miniature excitatory postsynaptic currents (mEPSCs), and the inhibition rate of SICs was significantly higher than mEPSCs with 30 μM ketamine (44.5 ± 3% inhibition vs. 28.3 ± 6% inhibition).

**Conclusion:**

Our data indicated that ketamine, not propofol and dexmedetomidine, within clinical concentration range inhibits glutamatergic transmission from astrocytes to neurons, which is likely mediated by the extrasynaptic GluN1/GluN2B receptor activation.

## Highlights

- Astrocytic glutamatergic activity is inhibited by ketamine at clinically relevant concentration.
- Synchronizations of astrocytic SICs are disrupted by ketamine.
- The same dose of ketamine inhibits SICs more obviously than mEPSCs.

## Introduction

Astrocytes, like neurons, are important participants in the brain’s integration and processing of information. Neuron-astrocyte network modulates advanced neural activities such as cognition (Yeh et al., 2015), emotion (Oliveira et al., 2015), motor (Acton et al., 2018), and sensory processing (Lopez-Hidalgo et al., 2017). Astrocytes release important neurotransmitters such as glutamate and GABA and participate in synaptic transmission (Araque et al., 2014). Besides, astrocytes express a variety of neurotransmitter receptors and perceive the activity of peripheral neurons (Pannasch and Rouach, 2013). So astrocytes interact with neurons dynamically and regulate pre-synaptic and post-synaptic activities. Recent studies have found that the activities of astrocytes are affected by general anesthetics (Thrane et al., 2012; Liu et al., 2016), but the effects of general anesthetics on the astrocyte-neuron transmission have not been systematically explored.

The glutamate that spontaneously released from astrocytes produces slow inward currents (SICs) via the extrasynaptic, GluN2B-containing NMDA receptors on neighboring neurons (Shigetomi et al., 2008). When compared with the miniature excitatory postsynaptic currents (mEPSCs), SICs exhibit significantly slower rise and decay times (Kovacs and Pal, 2017). SICs persists when neuronal and synaptic activity is suppressed, and both pharmacological and mechanical stimulation of astrocytes can induce SICs in neighboring neurons (Angulo et al., 2004). The physiological significance of SICs is to promote synchrony of neuronal activity, thus coordinating the activity of a brain area in a wider range. For example, in the thalamus, hippocampus and nucleus accumbens, researchers have found this extrasynaptic NMDAR response can occur synchronously in multiple neurons (Fellin et al., 2004; Pirttimaki et al., 2011).

Many studies of the mechanism of anesthesia believe that the loss of consciousness induced by general anesthetics is associated with a temporary breakdown of cortical functional connections, specifically the collapse of the synchronous activity pattern of cortical neurons, which makes the cortex unable to integrate information (Alkire et al., 2008; Lee et al., 2009; Rathmell and Wanderer, 2016). A single astrocyte makes about 100,000 synaptic connections with the surrounding neurons (Perea et al., 2014a), which synchronize the activity of neurons of the local brain area. So it is important to figure out the effects of anesthetics on the astrocyte-neuron activities.

In this work, we selected three anesthetics in clinical concentration: propofol (agonist of GABA_A_ receptor), ketamine (antagonist of NMDA receptor) and dexmedetomidine (agonist of α2-adrenergic receptors), which are agonist or antagonist of distinct receptors and all three drugs can induce hypnosis or loss of consciousness. The effects of the three distinct anesthetics on the astrocyte-derived SICs in acute cortical slices of rats were investigated. Our research may contribute to improving the understanding of the role of astrocytes in the mechanisms of loss of consciousness induced by general anesthetics.

## Materials and Methods

### Animals

All the experimental protocols were reviewed and approved by the Zunyi Medical University Animal Care and Use Committees (no. 2017-69 for pre-registration). According to the Guide for the Care and Use of Laboratory Animals published by the National Institutes of Health (Eighth Edition, 2011), the infant rats were reared with their mother in a specific pathogen-free animal room with controlled temperature (22–25°C) and a 12-hour light/dark cycle. All the rats had free access to a standard chow diet and purified drinking water.

### Slice Preparation

Coronal prefrontal cortex (PFC) slices (300 μm) were prepared from Sprague Dawley rats at postnatal day 21 (P21) to P40. Rats were anesthetized with chloral hydrate (10% wt/vol) and then perfused transcardially with ice-cold oxygenated (95% O_2_ / 5% CO_2_) high-sucrose solution containing (in mM) 2.5 KCl, 1.25 NaH_2_PO_4_, 2 Na_2_HPO_4_, 2 MgSO_4_, and 26 NaHCO_3_. The Rats were then decapitated to remove the brains. In a sectioning plate filled with ice-cold oxygenated high-sucrose solution, the isolated brain was sectioned at 300 μm using a vibratome (HM650V, Thermo, United States). After sectioning, the slices were incubated for 1 h at 34°C in an oxygenated artificial cerebrospinal fluid (ACSF) containing (in mM): 124 NaCl, 4.5 KCl, 2.0 CaCl_2_, 1.0 MgCl_2_, 1.2 NaH_2_PO_4_, 26 NaHCO_3_, 10 glycine, 10 glucose, pH 7.32.

### Electrophysiology

Glial cells and neurons were visually identified on an infrared-differential interference contrast optics (Olympus, Japan). Whole-cell patch-clamp recordings were performed from pyramidal neurons in the lateral prefrontal regions. For SICs recording, the internal solution contained (in mM): 135 CsCl, 8 NaCl, 4 Mg-ATP, 10 HEPES, 0.6 EGTA and 0.3 Na_2_-GTP, 310 mOsmol/L, pH 7.2. Slices were perfused in a chamber at 2–3 ml/min with an ACSF contained 100 micro molar picrotoxin (antagonist of gamma-aminobutyric acid A subtype receptor, GABA_A_) and 1 μM tetrodotoxin (TTX) (Angulo et al., 2004), which prevented the interference of action potential and inhibitory synaptic neurotransmission on the SICs recording. Whole-cell patch-clamp experiments were conducted with EPC10 amplifier (HEKA Elektronik, Germany). According to the nature of whole-cell recording, there is no randomization was performed.

### Pharmacology

In this experiment, the concentrations of the three general anesthetics were chosen based on their clinically effective ranges. For propofol, an agonist of GABA_A_ receptors, the plasma concentration to induce loss of consciousness in rat is around 10 μM (Yang et al., 1995); For ketamine, an antagonist of NMDA receptors, the plasma concentration of rats after intraperitoneal injection is around 30 μM (Ganguly et al., 2018); For dexmedetomidine, an agonist of alpha2-adrenergic receptors, the plasma concentration causing unconsciousness is around 40 nM (Plourde and Arseneau, 2017). The experimental concentrations of three drugs were 0.1, 1, 10, 20, and 40-fold of their clinically relevant concentrations. Propofol injectable emulsion (Diprivan, AstraZeneca, United States) was diluted to a final concentration in perfusion solution, while the perfusion solution containing the same concentration of emulsion (100 mg/mL soybean oil, 22.5 mg/mL glycerol, 12 mg/mL egg lecithin and 0.005% disodium edetate) was used as the control solution for experiments with propofol. Ketamine (Fujian Gutian Pharmaceutical Co., Ltd., China) and dexmedetomidine (Jiangsu Nhwa Pharmaceutical Co., Ltd., China) were diluted to experimental concentrations in perfusion solution.

In certain experiments, slices were treated with DHPG, an agonist of type I metabotropic glutamate receptors, to induce glutamate release from astrocytes (Porter and McCarthy, 1996). Some slices were treated with 1 mM fluorocitrate (a glia-specific metabolic inhibitor) for 1 h to inhibit astrocytic activity (Martín et al., 2007). Other antagonists included ifenprodil (5 μM), a GluN2B-specific NMDA receptor blocker; D(-)-2-Amino-5-phosphonopentanoic acid (D-AP5, 50 μM), a general NMDA receptor antagonist; tetanus neurotoxin (TeNT, 2 μM), which blocks the synaptic release of neurotransmitters; MK-801, 20 μM, a non-competitive antagonist of NMDA receptors; 2,3-Dihydroxy-6-nitro-7- sulfamoyl-benzo[f]quinoxaline (NBQX, 30 μM), a specific antagonist of non-NMDA (AMPA) glutamate receptors; TCN-201, 10 μM, a selective antagonist of GluN2A receptor.

### Astrocytes Culture

The dissociated subculture of cortical astrocytes was prepared and maintained as described previously (Zeng et al., 2008). Briefly, neonatal rat (1–2 days) was anesthetized and the prefrontal cortex was dissected surgically under sterile condition. The cortical tissue was cut and then incubated in 0.125% trypsin (Sigma, United States) at 37°C for 30 min, during which the trypsin was shaken once every 10 min. Then DMEM/F12 medium (Gibco, United States) containing 15% (v/v) fetal bovine serum was added to stop the action of trypsin. The cell suspension was centrifuged at 1000 rpm for 5 min to collect cells. The supernatant was discarded and the cells were suspended in complete medium containing DMEM/F12 and 15% (v/v) fetal bovine serum. Cells were planted in a flask and cultured in the incubator at 37°C in a humidified 5% CO_2_-95% air atmosphere. After 15 days, flasks were shaken (180 rpm) for 18 h and the medium was replaced by new ones. The subculture of cortical astrocytes was made by two times of digestion and centrifugation in the same procedure as mentioned above.

### Immunofluorescence

The subculture of astrocytes was planted in Petri dish containing DMEM/F12 complete medium and cultured for 24 h. Astrocytes were fixed in 4% paraformaldehyde for 30 min, washed with 0.01 mol/L phosphate-buffered saline (PBS) three times, permeated by 0.02 % TritonX-100 for 3 min, washed with 0.01 mol/L PBS three times. Astrocytes were incubated with rabbit anti-GFAP (1:1000, Abcam, United States) at 4°C overnight, followed by incubations with FITC-conjugated goat anti-rabbit IgG (1: 1000 in PBS, Sigma, United States) for 90 min. At last, DAPI (1:2000, Abcam) was applied for 5 min to label the nuclei of all astrocytes. After staining, Petri dishes were examined using a Leica SP2 confocal microscope (Leica, Germany).

### Analysis of Extracellular Glutamate Concentration

The concentration of extracellular glutamate was identified using HPLC with a fluorescence detector. HPLC was performed based on the methods previously published with some adjustment (Zeng et al., 2008) and the tester was blinded to the procedure of supernatant harvest. The HPLC system (1260 Infinity, Agilent, United States) comprised of a quaternary pump system (G1311B), fluorescence detector (G1321B, λex = 337 nm, λem = 457 nm), HPLC chemo-station, a 5 μm biophase octadecylsilyl and analytical column (150 mm × 4.6 mm). All astrocytes were seeded in 6-well plates and employed for individual tests when astrocytes produced a confluent layer 5 days after seeding. At this stage, the culturing medium was discarded and washed two times with 0.01 PBS, after that incubated with the free-serum medium. 10 μM DHPG (made with 0.01 M PBS) was added to the various wells and supernatant was harvested at 2, 4, 6, 8, and 10 min, respectively. Supernatants were derivatized with O-phthalaldehyde/beta-mercaptoethanol (OPA) for 2 min prior to injection. The mobile phase was composed of 0. 01 M Na2HPO4, 0.1 mM EDTA and 30% methanol. The system was operated at 38°C with a flow rate of 1 mL/min. The calibration curves and quantifications were deduced on the maximum areas calculated with Agilent Lab Advisor software. Control experiments were performed with adding 0.01 M PBS to wells after which supernatants were harvested to determine the basal amount of glutamate. Effects of the three anesthetics (diluted to 0.1, 1, 10, and 100-fold of their clinically relevant concentrations in 0.01 M PBS) were always compared with the controls performed on the same culture preparation. The same experiments were repeated in five different astrocyte cultures.

### Data Collection and Analysis

Patchmaster 2.0 software (HEKA Elektronik, Germany) was used for data acquisition, while data analysis was performed by Spike 2 software (CED, ENGLAND). For recording SICs and mEPSC, voltage-clamp traces were recorded at a holding potential of -60 mV. The noise level of mEPSC was set as 5 pA while SICs as 30 pA. Only stable current traces that exhibit a single peak distribution in the on-line histogram analysis were used for subsequent analysis. Average amplitude and frequency of SICs and mEPSC were calculated from 30 min long current traces before and after drug application. The series resistance was continuously monitored during the recording and was accepted when the series resistance of the current was less than 30 Ω and with less than 10% change. The amplitude of the SICs is the difference between the SICs peak and the average of the current baseline within the 20 ms before the appearance of the SICs. The zero to one hundred percent rise time and 10 to 90% decay time for SIC events were calculated with single exponential fits. Power And Sample Size software was used to predetermine the sample size of recorded neurons. All data were presented as mean ± SD. Statistical analysis of two groups was performed with Student’s *t*-test by utilizing GraphPad PRISM 6 Software (GraphPad Software Inc., United States). When multiple groups were compared, statistical analysis was performed with one-way analysis of variance (ANOVA) followed by Brown-Forsythe test. Cumulative probability curves for SICs rise times and decay times were tested by Kolmogorov-Smirnov test. Statistical significance at the *p* < 0.05 level were considered statistically significant.

## Results

### SICs Is Generated by Glutamate Release From Astrocytes

**FIGURE 1:**
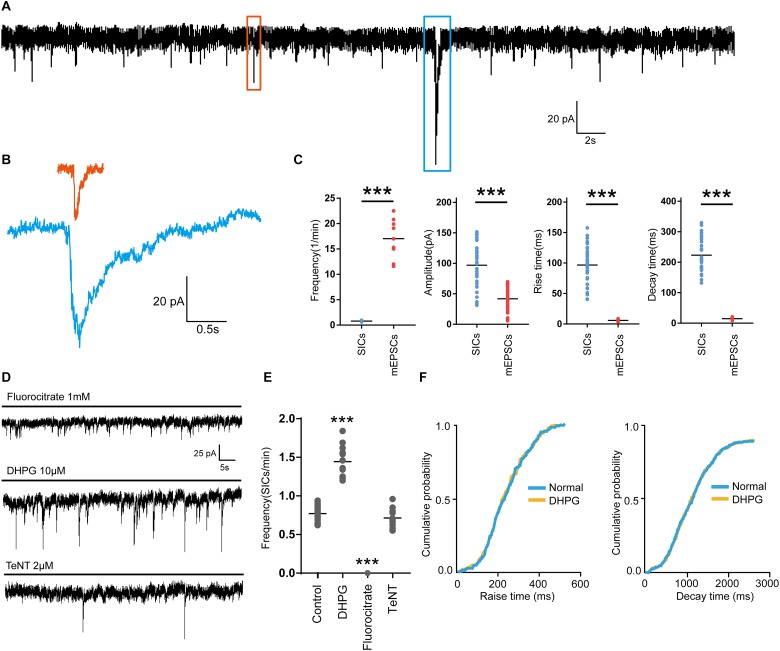
Parameters of SICs and mEPSC from the prefrontal cortex neuron. **(A)**, Representative trace of SICs and mEPSCs recorded from a PFC neuron at a holding potential of –60 mV. The red square marks a mEPSC and a SIC is labeled by the blue square. **(B)** mEPSC (red) and SIC (blue) were showed in a larger scale. **(C)** Statistical summary of frequency, amplitude, rise and decay time of SICs and mEPSCs. Differences with statistically significant (^∗∗∗^*p* < 0.001, compared with control) were verified by unpaired *t*-test. **(D)** Representative traces of SICs exposed to fluorocitrate, DHPG and TeNT administration. **(E)** Statistical summary of frequency of SICs recorded in 10 min before and after fluorocitrate, DHPG and TeNT administration. **(F)** Cumulative probability curves for SIC rise times and decay times for normal (blue line) and DHPG treatment (yellow line), no statistical difference, Kolmogorov-Smirnov test. Data were presented as mean ± SD. Dots represent individual data.

First, we investigated the presence of SICs on PFC neurons. When we held PFC neurons near their resting membrane potential (-60 mV) in magnesium-free ACSF (as MgCl_2_ was not administered to the solution), which prevent the voltage-dependent block of glutamate receptors of the NMDA subtype, and 1 μM TTX was applied into the ACSF to block action potential propagation in the neuronal network, we observed spontaneous SICs in 89% of the recorded neurons (63 neurons from 20 rats) with comparable parameters to literature data (Pirttimaki and Parri, 2012). The average frequency of SICs was 0.78 ± 0.16/min, with the amplitude of 96.9 ± 37.9 pA, a rise time of 96.8 ± 30.5 ms and a decay time constant of 269.5 ± 32.8 ms (38 events from 22 neurons, Figure 1A,C). The parameters of SICs can be unambiguously separated from miniature excitatory postsynaptic currents (mEPSCs) (Figure 1A,B). The decay time constant of mEPSCs were 25.68 ± 2.6 ms (45 events from 9 neurons, Figure 1B,C), which were two magnitudes faster than SICs, thus SICs are clearly distinguishable. We then sought evidence to prove whether the neuronal SICs generation could be affected by pharmacological manipulations known to inhibit or activate astrocytes in PFC slices. First, the slices were treated with 1 mM fluorocitrate for 1 h prior to recording. This drug, a specific blocker of astrocytes, inhibits the Krebs cycle of astrocytic metabolism (Largo et al., 1996). Under the same experimental arrangement described above, no SICs was detected from fluorocitrate incubated slices (12 neurons from 3 rats, Figure 1D,E). Second, when 10 μM DHPG (agonist of astrocytic type I metabotropic glutamate receptors) were bath applied for pharmacological activation of astrocytes (Porter and McCarthy, 1996), we observed a significant increase in the frequency of the SICs (Figure 1D,E). The 10–90% rise and decay time of the slow currents recorded before and during DHPG showed no different (Figure 1F). Furthermore, SICs were still present after slice incubation (2 h) with 2 μM tetanus neurotoxin (TeNT) (Figure 1D,E), which blocks the synaptic release of neurotransmitters (Link et al., 1992). Altogether, the above observations strongly suggest that SICs are of the astrocytic origin.

### The Astrocytic Glutamatergic Transmission Was Inhibited by Ketamine at a Clinically Relevant Concentration

**FIGURE 2:**
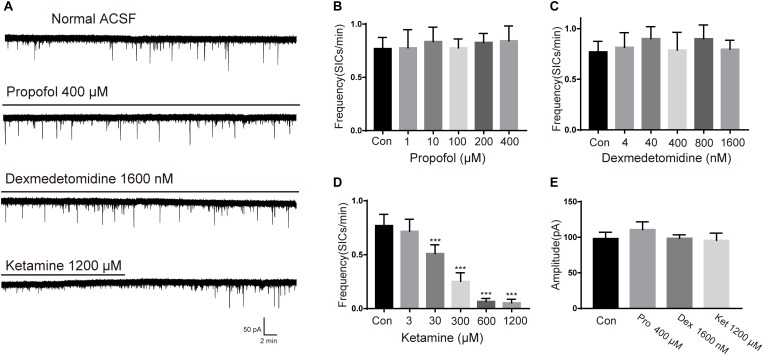
Effects of propofol, dexmedetomidine and ketamine on SICs. **(A)** Example large scale traces of SICs exposed to the highest concentration of propofol, dexmedetomidine and ketamine. **(B–D)** bar graphs illustrate SICs frequency affected by three anesthetics with different concentrations. **(E)** Statistical summary of amplitude of SICs affected by three anesthetics with the highest concentration in this study. Differences with statistically significant (^∗∗∗^*p* < 0.001, compared with control) were verified by one-way ANOVA followed by Brown-Forsythe test. Data were presented as mean ± SD. Con = control, Pro = propofol, Dex = dexmedetomidine, Ket = ketamine.

To study the effects of different general anesthetics on the astrocytic glutamatergic transmission, the selected anesthetics were added to oxygenated ACSF for a slice pre-incubation (15 min), the solution containing anesthetic were continuously perfused during whole-cell patch-clamp recordings. Due to the different preparation protocols of the 3 drugs (see section “Materials and Methods”), SICs recording in slices under ASCF containing emulsion was taken as the control for propofol, while SICs recording under normal ASCF was taken as the control for both ketamine and dexmedetomidine. The concentrations tested for each general anesthetic were chosen as 0.1, 1, 10, 20, and 40-fold of their clinically relevant concentrations (see section Materials and Methods). For propofol, the test concentrations were set as 1, 10, 100, 200, and 400 μM. Propofol with all selected concentrations showed no significant effect on the amplitude or frequency of SICs (Figure 2B,E). Dexmedetomidine with selected concentrations (4, 40, 400, 800, and 1600 nM) were also found ineffective in changing the amplitude or frequency of SICs (Figure 2C,E), indicating propofol and dexmedetomidine exert no effect on the astrocytic glutamatergic transmission within the clinically relevant concentration range. For ketamine, the test concentrations were 3, 30, 300, 600, and 1200 μM. We found that the frequency of SICs dropped to 0.53 ± 0.18 SICs/min at a concentration of 30 μM from 0.77 ± 0.16 SICs/min in control group (10 neurons from 5 slices, Figure 2D). The inhibition of the frequency of SICs reached 0.35 ± 0.15 SICs/min at 300 μM (8 neurons from 3 slices, Figure 2D). Ketamine with 600 (5 neurons from 3 slices) and 1200 μM (5 neurons from 3 slices) inhibited the frequency of SICs to 0.087 ± 0.06 and 0.083 ± 0.05%, respectively (Figure 2D). Ketamine with all selected concentrations did not change the amplitude of SICs (Figure 2E). When washed out for 15 min, the frequency of SICs recovered to about 80% (Figure 2A, the bottom trace) of the control level, demonstrating that the inhibition induced by ketamine is largely reversible.

### Synchronization of Astrocytic SICs Was Inhibited by Ketamine

**FIGURE 3:**
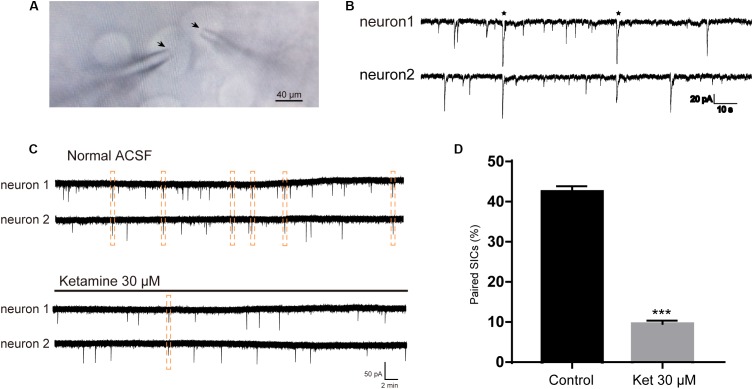
Ketamine inhibit synchronized SICs from pair-recorded PFC neurons. **(A)** Infrared chromatic aberration photo of pair-recorded PFC neurons. Tips of glass microelectrodes were marked with arrow. **(B)** Example traces showed synchronized SICs in two different PFC neurons recorded simultaneously. Two pairs of synchronized SICs were marked by asterisks. **(C)** Example large scale traces of synchronized SICs exposed to normal ACSF and 30 μM ketamine, synchronized SICs were marked by dashed box. **(D)** Statistical summary of the occurrence rate of paired SICs. Differences with statistically significant (^∗∗∗^*p* < 0.001, compared with control) were verified by unpaired *t*-test. Data were presented as mean ± SD.

SICs can occur simultaneously in adjacent pyramidal neurons, which promote synchrony of neuronal activity (Perea et al., 2014a). In the next series of experiments, we sought evidence whether ketamine can affect the synchronization of astrocytic SICs. Since propofol and dexmedetomidine at clinically relevant concentration showed no inhibitory action on the frequency of SICs, we did not examine their effects on SICs synchronization. Pairs of PFC neurons were patched where somata were within 20 μm (Figure 3A), and spontaneous activity was recorded in parallel in voltage clamp mode in a recording period of 10 min. When the delay between the onset of one SIC recorded in one neuron and the other SIC appearing in the paired neuron was <0.05 s, count as a valid synchronization of SICs (Figure 3B). We first record six pairs of PFC neurons distant by <20 μm under normal ACSF, during 10 min period, the occurrence rate of synchronized SICs was 42.5 ± 1.4% (68 SICs were recorded from 12 neurons) (Figure 3C, up panel and Figure 3D). Then we recorded 6 pairs of PFC neurons distant by <20 μm under ACSF containing 30 μM ketamine, the occurrence rate of synchronized SICs dropped to 9.6 ± 0.8% (36 SICs were recorded from 12 neurons) (Figure 3C, bottom panel and Figure 3D). The occurrence rate of synchronized SICs was 0 when 300 μM ketamine were tested (5 SICs were recorded form 2 pairs of PFC neurons). These data thus indicate that ketamine at clinically relevant concentrations exerts a significant inhibitory effect on the astrocyte-mediated SICs synchronization.

### Ketamine Exerts No Effects on the Astrocytic Glutamate Release

**FIGURE 4:**
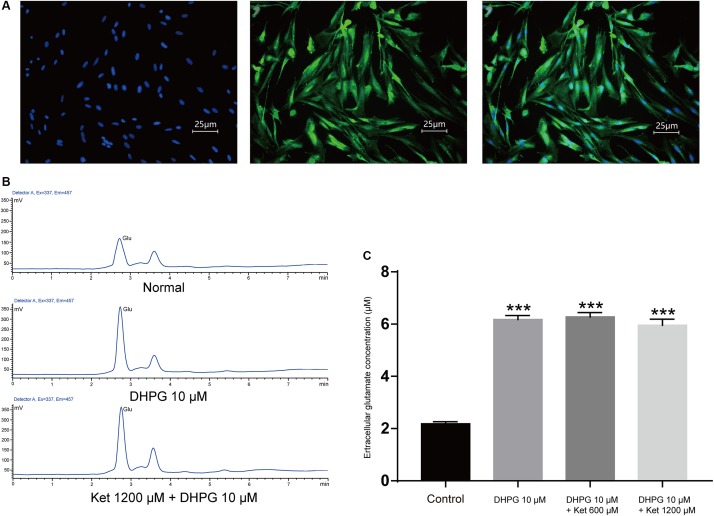
Effect of ketamine on glutamate release from cultured prefrontal cortex astrocytes. **(A)** Micrographs of cultured prefrontal cortex astrocytes labeled for DAPI (left), GFAP (middle) their overlay (right). **(B)** HPLC chromatogram of glutamate in astrocyte culture medium after DHPG (middle) or ketamine (down) administration. **(C)** Statistical summary of the glutamate concentration. Differences with statistically significant (^∗∗∗^*p* < 0.001, compared with control) were verified by one-way ANOVA followed by Brown-Forsythe test. Data were presented as mean ± SD. Glu = glutamate, Con = control, Ket = ketamine.

SICs are due to the spontaneous release of glutamate from astrocytes, thus the inhibitory actions of ketamine on SICs might be consequences of the decrease of glutamate release from astrocytes caused by ketamine. In order to investigate this possibility, we next explore whether ketamine inhibits DHPG-evoked glutamate release from cultured PFC astrocytes. PFC astrocytes were identified with immunofluorescence stained by mouse GFAP antibody (Figure 4A). The immunohistochemical staining after two generations showed that the purity of the astrocytes was more than 95% (data not shown). Glutamate efflux from astrocyte cultures was assayed with HPLC. The base level of glutamate release from cultured prefrontal cortex (PFC) astrocytes was 2.18 ± 0.84 μM (Figure 4B,C; five wells). Application of 10 μM DHPG for 10 min increased the concentration of glutamate to 6.18 ± 0.15 μM (Figure 4B,C; of five wells). Pre-incubation of ketamine (300 and 1200 μM) for 10 min could not influence the DHPG-induced glutamate efflux (5.96 ± 0.23 μM for ketamine 1200 μM + DHPG 10 μM; Figure 4B,C; of five wells), which suggest that DHPG-induced astrocytic glutamate release is not affected by ketamine.

### Ketamine-Induced Inhibition of SICs Is Mediated by GluN1/GluN2B Receptors

**FIGURE 5:**
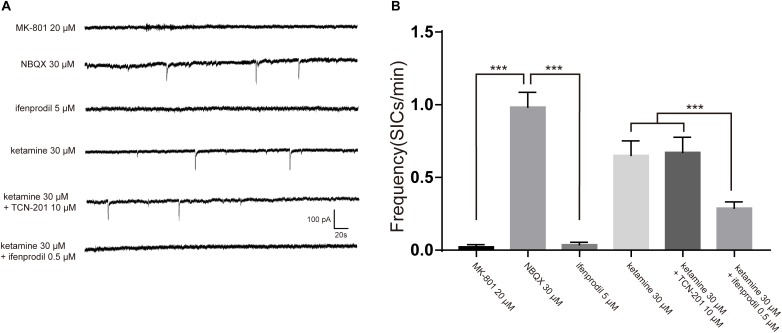
The inhibition of SICs by ketamine was enhanced by selective antagonist of GluN1/GluN2B receptor. **(A)** Example traces of SICs with different antagonists administration. **(B)** Statistical summary of SICs frequency affected by different antagonists. Differences with statistically significant (^∗∗∗^*p* < 0.001, compared with control) were verified by one-way ANOVA followed by Brown-Forsythe test. Data were presented as mean ± SD.

Negative results with glutamate assay of astrocyte cultures raised the possibility that ketamine-induced inhibition of SICs was due to blockade of NMDA receptors. As ketamine is a non-selective antagonist for the NMDA receptor (Lord et al., 2013), we need to seek the NMDA receptor subtype responsible for SICs. The effects of different antagonists on SICs were present in Figure 5A. In the presence of 20 μM MK-801, a non-competitive antagonist of NMDA receptors, the frequency of SICs were dropped to 0.02 ± 0.01/min (Figure 5B, 10 neurons). However, spontaneous SICs were observed with normal frequency (0.98 ± 0.10/min) in 30 μM NBQX (Figure 5B, 5 neurons), a specific antagonist of non-NMDA (AMPA) glutamate receptors. Then we perfused selective antagonist of GluN1/GluN2B receptor, ifenprodil (5 μM) (Bettini et al., 2010), which inhibited the frequency of SICs significantly (Figure 5B, 10 neurons). We also found that 0.5 μM ifenprodil, not 10 μM TCN-201 [selective antagonist of GluN1/GluN2A receptor (Edman et al., 2012)] could significantly enhance the inhibitory effect of 30 μM ketamine on the frequency of SICs (from 0.65 ± 0.10/min to 0.29 ± 0.04/min, Figure 5B, 10 neurons). Such results suggest that the ketamine-induced inhibition of the frequency of SICs is due to the blockade of GluN1/GluN2B receptors by ketamine.

### Astrocytic SICs Are More Sensitive to Ketamine Than Synaptic mEPSCs

**FIGURE 6:**
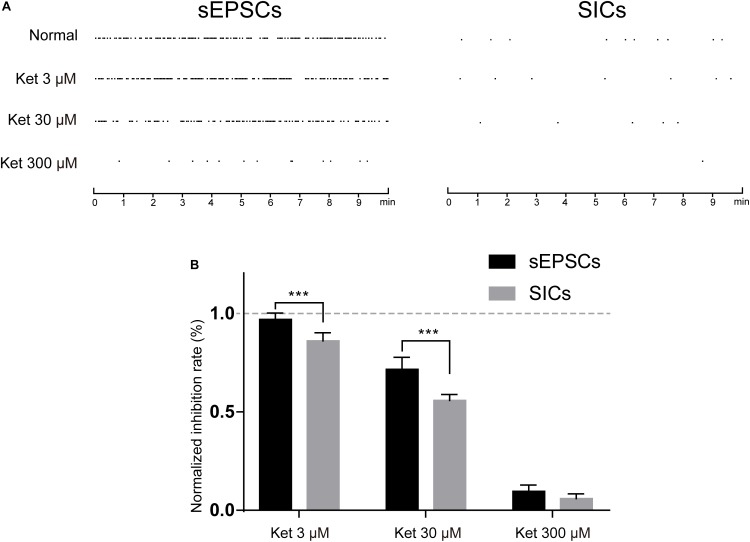
The inhibitory effects of ketamine on mEPSCs and SICs. **(A)** events marker of mEPSCs (left) and SICs (right) in a representative 10 min period. Ketamine inhibited both mEPSCs and SICs in a concentration dependent manner. **(B)** Normalized inhibition rate of ketamine on mEPSCs and SICs. Differences with statistically significant (^∗∗∗^*p* < 0.001, compared with control) were verified by two-way ANOVA with post tests. Data were presented as mean ± SD.

Depending on the subunit composition, GluN1/GluN2A and GluN1/GluN2B receptors have distinct pharmacological and kinetic properties. GluN1/GluN2A receptor is a target of glutamate released from a synaptic source, which is characterized by rapid kinetics (mEPSCs) (Nakanishi et al., 1998). While extrasynaptic GluN1/GluN2B are activated by glutamate released from a non-synaptic (astrocytic) source, which is characterized by slow kinetics (SICs) (Angulo et al., 2004). Different in the source of glutamate and kinetic properties raised the possibility that ketamine may exert a different effect on the fast (synaptic) and slow (non-synaptic) glutamatergic transmission. In order to investigate this possibility, we compared the effect of 3, 30, and 300 μM ketamine on the GluN1/GluN2A and GluN1/GluN2B mediated currents. mEPSCs and SICs were recorded and distinguished by their frequency and decay time as stated above. The frequency of mEPSCs was 22.7 ± 3.4/min in control, and 22.1 ± 3.2/min with 3 μM ketamine, 16.9 ± 1.5/min with 30 μM ketamine, 2.3 ± 0.2/min with 300 μM ketamine. The frequency of SICs was 0.85 ± 0.12/min in control, and 0.72 ± 0.12/min with 3 μM ketamine, 0.48 ± 0.10/min with 30 μM ketamine, 0.06 ± 0.001/min with 300 μM ketamine (Figure 6A). In Figure 6B, inhibition rate of 3, 30, and 300 μM ketamine on mEPSCs and SICs were presented, which indicated that ketamine at low concentration (3 μM) could inhibit the frequency of SICs, but not mEPSCs. And, the inhibition rate of SICs was significantly higher than mEPSCs with 30 μM ketamine (44.5 ± 3% inhibition vs. 28.3 ± 6% inhibition). Taken together, one might conclude that GluN1/GluN2B mediated astrocytic excitatory current is more sensitive to ketamine.

## Discussion

In the present study, we demonstrated that ketamine significantly suppressed GluN1/GluN2B receptor-induced SICs in acute rats brain slice, in a concentration-dependent manner. On the contrary, propofol and dexmedetomidine did not affect SICs, which represent glutamatergic transmission from astrocytes to neurons. Three anesthetics were tested in the ranges that correspond to their respective plasma concentrations during anesthesia. Ketamine inhibited the frequency of SICs by more than 30% when used at 30 μM, which is around the plasma concentration of rats after intraperitoneal injection (Ganguly et al., 2018). Propofol and dexmedetomidine had no effect on the frequency of SICs, even at 40-fold of their clinically relevant concentrations (Franks, 2008; Plourde and Arseneau, 2017).

Connexins form functional gap junction channels that enable electrochemistry communication between adjacent astrocytes and neurons (Nagy and Rash, 2000). Early experiment (Liu et al., 2016) exploring anesthetic mechanism in astrocytes compared the effects of propofol, ketamine, and dexmedetomidine on the connexin channel functions of astrocytes. They reported that propofol was found to inhibit gap junction within clinically relevant ranges (100–150 μM), either ketamine nor dexmedetomidine had an effect on gap junction channels within clinically relevant ranges. Combined with our results, one can infer that both propofol and ketamine, at clinically relevant concentrations, are an effective compound in affecting the activities of astrocytes, however, they aimed at different targets. Propofol may inhibit the functional astrocytic network by inhibiting the activity of connexin channel and decreasing calcium signaling between astrocytes (Parys et al., 2010). While ketamine inhibits the glutamate transmission from astrocytes to neurons, thereby inhibiting the astrocytic regulation of neurons, especially the synchronous activity of nearby neurons (Fellin et al., 2004).

In this study, we found direct evidence for the inhibition of astrocyte-mediated SICs synchronization by ketamine at clinical concentrations. A significant 78% inhibition of the occurrence rate of synchronized SICs on paired PFC neurons was produced by 30 μM ketamine. Astrocytes play a key role for the synchrony of regional neurons, stimulation of one astrocyte can activate other astrocytes in the region and trigger continuous depolarization and high-frequency discharge of adjacent neurons (change and maintain new patterns of neuronal activity) (Perea et al., 2014a). Under the control of astrocytes, the cortex forms a wide range of neuronal clusters that receive and output special signals, and eventually from the neuronal basis to encode and integrate advanced neural information.

Astrocytes can release glutamate through calcium-dependent and calcium-independent mechanisms, such as vesicular release (Yaguchi and Nishizaki, 2010), the reverse operation of glutamate transporters (Yoshizumi et al., 2012) and the opening of hemichannels (Ye et al., 2003). Our present research did not focus on the specific mechanism by which astrocytes release glutamate. However, bath applications of DHPG were shown previously to trigger intracellular calcium transients in astrocytes (Muyderman et al., 2001). So DHPG increases the release of glutamate from astrocyte cultures support the involvement of a calcium-dependent release mechanism. Our results of glutamate assay showed that ketamine with 10-fold of the clinical concentration exerts no effects on the astrocytic glutamate release, which indicate that calcium-independent vesicular pattern of astrocytic glutamate release is not a potentially important mechanism in ketamine-induced inhibition of glutamatergic transmission between neurons and astrocytes. However, identifying the actual effects of ketamine or other anesthetics on the glia release glutamate *in vivo* animal model still need additional experiments.

Our work demonstrated that SICs are consequences of astrocytic activity. Stimulation of astrocytes by group I metabotropic glutamate receptor agonist led to the appearance of SICs, and inhibition of astrocytes by fluorocitrate prevented the development of these events. The astrocytic source of SICs was verified by optogenetic activation of astrocytes (Perea et al., 2014b), a more advanced technology allowing specific activation of astrocytes in slice preparations. Our results showed that SICs were prevented by ketamine or GluN1/GluN2B receptors antagonists, which indicates that ketamine-induced inhibition of SICs is mediated by the extrasynaptic NMDA receptors containing NR2B subunits. However, it can be a matter debate whether GluN1/GluN2B receptors involved in the astrocyte-neuron modulation of ketamine are neuronal extrasynaptic or astrocytic receptors because functional NMDA receptors were found presenting on cortical astrocytes (Dzamba et al., 2013). As the SICs-blocking effects of ketamine were very similar to the inhibition caused by ifenprodil, we cannot clearly exclude the possibility that astrocytic NMDA receptors contribute to the ketamine-inhibition of SICs. Furthermore, based on the facts that astrocytes express functional AMPA receptors (Matthias et al., 2003) and ketamine is an indirect AMPA receptor agonists (Krystal et al., 2013), one may hypothesize that ketamine potentiates the glutamatergic AMPA transmission while inhibits SICs. We did not add selective AMPA antagonist when evaluating the effects of ketamine on SICs, thus the low concentration of ketamine was insufficient to inhibit mEPSCs may due to the ketamine-induced potentiation of glutamatergic AMPA transmission.

To the best of our knowledge, our present work first found direct evidence prove that astrocyte-neuron glutamatergic transmission, which represented by SICs, were inhibited by ketamine in clinical concentration. The effects of general anesthetics on non-neuronal cells and the potential mechanism by which these drugs induce unconsciousness are not widely appreciated. Most previous studies focused on the effects of general anesthetics on the neuronal injury related to intracellular calcium homeostasis (Mantz et al., 1994; Yang et al., 2008) or glutamate uptake in astrocytes (Miyazaki et al., 1997; Sitar et al., 1999). One study explored the conscious targets of anesthetics (including ketamine) in astrocytes (Thrane et al., 2012). Combined with our findings, it is important that direct suppression of astrocytic activities (such as synchronized calcium signals and SICs) by anesthetics were found as a potential mechanism under their unconsciousness effects. In addition, the previous study reported that anesthetic doses of isoflurane are insufficient to affect neuronal responses but sufficient to obviously inhibit sensory-evoked astrocyte calcium transients (Schummers et al., 2008). Their results are in line with our present finding showed that low concentration of ketamine was insufficient to inhibit mEPSCs but sufficient to inhibit astrocytic SICs. All these studies indicate that, compared with neurons, astrocytes activities are more vulnerable to anesthetics. So the inhibition of astrocytic function may play a key role in the early stage of unconsciousness induced by general anesthetics.

Ketamine at sub-anesthetic doses acts as a fast-acting antidepressant (Krystal et al., 2013). The underlying mechanisms involve activating the mammalian target of rapamycin (mTOR) pathway leading to improvement of synaptic signaling (Li et al., 2010), actions on the glutamatergic system, 5-HT system and dopaminergic system (De Gregorio et al., 2018), improving synaptogenesis (Liu et al., 2012) and neurotrophic function (Abdallah et al., 2015). Thus the ability of ketamine to modulate the SICs at sub-anesthetic doses in its antidepressant effects needs to be discussed. Many factors of stress response such as immunologic attack, increased levels of reactive oxygen species and reduced levels of free radical scavengers lead to astrocytes loss (Banasr and Duman, 2008). Because astrocytes are centrally involved in glutamate inactivation, astrocytes loss may elevate glutamate levels in both synaptic and extrasynaptic spaces. The astrocytic origin of SICs was confirmed by our results, thus the inhibition of astrocytic SICs by ketamine may depress glutamate neurotransmission when extracellular glutamate level was elevated by astrocytes loss, which in turn compromising functional astrocyte–neuron connectivity during the depression. Moreover, the blockade of extrasynaptic NMDA receptors also appears to be critical to the antidepressant effects of ketamine (Krystal et al., 2013), and we proved that ketamine inhibited SICs by blocking the extrasynaptic NMDA receptors containing NR2B subunits supporting such theory.

## Conclusion

In summary, the present findings suggest that by blocking GluN1/GluN2B receptors, ketamine have an inhibitory role in astrocytic glutamatergic transmission. The decrease of astrocyte-mediated SICs synchronization due to ketamine administration at clinical concentration may have serious implications for the development of dissociative cognitive impairment during ketamine anesthesia.

## Author Contributions

YZ, SW, and LX analyzed the data and drafted the manuscript. CL, SY, LZ, and WZ collected the data. TY approved the final version of the manuscript.

## Conflict of Interest Statement

The authors declare that the research was conducted in the absence of any commercial or financial relationships that could be construed as a potential conflict of interest.
